# Effects of the Prenatal Maternal Health Status on Calf Disease Prevalences and Respective Genetic Parameter Estimates in German Holstein Cattle

**DOI:** 10.1111/jbg.12906

**Published:** 2024-10-27

**Authors:** Laura Aufmhof, Tong Yin, Katharina May, Sven König

**Affiliations:** ^1^ Institute of Animal Breeding and Genetics Justus‐Liebig‐University Gießen Gießen Germany

**Keywords:** genotype x prenatal maternal health status interactions, health traits, intergenerational transmission

## Abstract

The aim of the present study was to infer phenotypic responses and genetic parameters of the F1 calf diseases diarrhoea (DIAR) and pneumonia (PNEU) in dependency of the prenatal maternal health status (PMHS) of the dam and of the herd‐calving year. The PMHS considered diagnoses for the cow disease mastitis (MAST) and claw disorders (CD) during gestation of F0 dams. Furthermore, 305‐d milk production traits of F1 offspring from either healthy or diseased dam groups were compared. The study comprised 20,045 female calves (F1 = generation 1) and their corresponding dams (F0 = parental generation 0), kept in 41 large‐scale herds. All F1 calves were from their dams' 2nd parity, implying that all dam (maternal) diseases were recorded during the first lactation and dry period of the dams. The F1 calves were phenotyped for DIAR up to 30 days post‐partum, and for PNEU up to 180 days of age. At least one entry for the respective disease implied a score = 1 = sick, otherwise, a score = 0 = healthy, was assigned. Production records of the 10,129 F1 cows comprised 305‐d records in first lactation for milk yield (MY), protein yield (PY) and fat yield (FY). Linear and generalised linear mixed models were applied to infer phenotypic responses of F1 traits in dependency of the PMHS for CD and MAST. A diagnosis for MAST or CD in F0 cows during gestation was significantly (*p* ≤ 0.05) associated with an increased prevalence for DIAR and PNEU, with pairwise differences of least‐squares‐means between calves from healthy and diseased cow groups up to 3.61%. The effects of PMHS on 305‐d production traits in offspring were non‐significant (*p* > 0.05). In bivariate genetic analyses, DIAR and PNEU were defined as different traits according to the PMHS, i.e., DIAR‐MAST_healthy_ and DIAR‐MAST_diseased_, DIAR‐CD_healthy_ and DIAR‐CD_diseased_, PNEU‐MAST_healthy_ and PNEU‐MAST_diseased_, and PNEU‐CD_healthy_ and PNEU‐CD_diseased_. The direct heritabilities for DIAR and PNEU were quite similar in the healthy and respective diseased dam group. Slightly larger direct heritabilities in the diseased dam groups were due to increased genetic variances. Maternal heritabilities were quite stable and smaller than the direct heritabilities. In random regression models, genetic parameters for DIAR and PNEU were estimated along the continuous herd‐calving‐year prevalence scale, considering a prevalence for MAST and CD (based on the 20,045 dam records plus 16,193 herd contemporary records) in the range from 0% to 30%. Direct heritabilities for PNEU were quite stable along the herd‐calving‐year gradient for MAST and CD. For DIAR, we observed stronger estimate fluctuations, especially increasing direct heritabilities in dependency of the herd‐calving‐year prevalence for MAST from 0.13 (at a MAST prevalence of 0%) to 0.30 (at a MAST prevalence of 30%). Consequently, obvious genotype x herd‐calving‐year PMHS interactions were observed for DIAR on the prenatal MAST scale, with a minimal correlation of 0.48 between direct genetic effects at 0% MAST prevalence and at 30% MAST prevalence. The correlations between direct genetic and maternal genetic effects were antagonistic at all herd‐calving‐year prevalence levels, displaying strongest fluctuations for “DIAR‐MAST.” The genotype x herd‐calving‐year PMHS interactions for DIAR suggest consideration of specific sires according to the herd health status for CD and for MAST.

## 1 Introduction

Diarrhoea (**DIAR**) and pneumonia (**PNEU**) are economically important diseases contributing to calf mortality up to an age of six months (e.g., Svensson, Linder, and Olsson 2006; Johnson, Burn, and Wathes 2011). Medrano‐Galarza et al. (2018) and Dachrodt et al. (2021) reported a prevalence for DIAR and for PNEU larger than 20%. The aetiology of DIAR and PNEU is multi‐factorial including the farm management (Svensson et al. 2003), bacterial and viral pathogen activities (Van der Fels‐Klerx et al. 2001; Muktar et al. 2015), and genetic effects. The heritabilities for DIAR and PNEU ranged from 0.04 (Gonzalez‐Peña et al. 2019) to 0.22 (Snowder et al. 2005; Neibergs et al. 2014). In addition, environmental factors might interact with genetic mechanisms, contributing to possible genotype x environment (**G x E**) interactions for health traits. Shabalina et al. (2021) proved G x E interactions for the cow health disorders mastitis and dermatitis digitalis due to differences in German organic and conventional production system characteristics. In contrast, in Austrian dairy cattle, Pfeiffer et al. (2016) detected no severe genotype by production system interactions for functional traits including clinical mastitis, early fertility disorders, cystic ovaries and milk fever. For calf respiratory diseases, Haagen et al. (2021) indicated that herd‐by‐sire interactions accounted for only less than 1% of the phenotypic trait variation.

In addition, prenatal environmental conditions were associated with calf disease susceptibility, which is termed “intergenerational transmission” (David et al. 2019). Intergenerational transmission means the direct exposure of the F0 parental generation to a stressor and its effect on the F1 germ cell or foetus (Klengel, Dias, and Ressler 2016; David et al. 2019). To date, most studies addressing intergenerational transmission effects in dairy cows focused on in utero heat stress in relation to growth, health and production in the offspring (e.g., Guo et al. 2016; Monteiro et al. 2016). In utero heat stress during late gestation impaired immune functions of calves during the first 28 days after birth (Tao et al. 2012). In genetic time‐lagged heat stress studies, Kipp et al. (2021b) found alterations of genetic parameters for cow health and production traits in the F1 generation due to heat stress during late pregnancy of F0 dams. Accordingly, Kipp et al. (2021b) postulated possible epigenomic effects. Skibiel et al. (2018) showed that epigenetic effects are the main cause of intergenerational transmission, e.g., specific DNA methylations due to in utero heat stress. With regard to the early calf diseases DIAR and PNEU, Seeker et al. (2018) and Yin, Halli, and König (2022) indicated strong involvement of regulatory genes for immune defence mechanisms and the role of possible epigenetic processes. Such epigenetic modifications might contribute to genetic parameter alterations of offspring diseases, as shown by Yin, Halli, and König (2022) for PNEU on a continuous prenatal climate scale. Random regression models (**RRM**) have been applied for the estimation of genetic parameters in dependency of herd characteristics, including the average herd production or BCS level (Calus and Veerkamp 2003), herd size and sire origin (Yin and König 2018a), or herd disease prevalence (Twomey et al. 2018). Alterations of genetic (co)variance components on continuous herd gradients might be due to the activation of genes under specific environmental conditions (Schaeffer 2004).

Abuelo (2020) and Vlasova and Saif (2021) associated metabolic and immune dysregulation during gestation with calf diseases in the F1 generation. Furthermore, abnormal prenatal nutrition due to dam food restriction can impair the F1 health status and F1 productivity (Breier 2006). Heat stress and malnutrition were key drivers contributing to the occurrence of diseases in dairy cows, with long‐lasting effects across generations (Ingvartsen and Moyes 2013; Lemal et al. 2023). Thus, we hypothesise direct dam diseases—calf disease associations, i.e., intergenerational transmission effects on the offspring health status due to infection diseases in dams. Udder and claw diseases of dairy cows induced strong detrimental time‐lagged effects on production and other health traits during the ongoing lactation (Krishnamoorthy et al. 2021; Vanhoudt et al. 2021). Carvalho et al. (2020) indicated a lower risk for DIAR of calves from diseased dams compared to F1 offspring from the healthy dam control group. Hence, time‐lagged effects can be extended to an intergenerational perspective, contributing to possible genotype x prenatal maternal health status (**G x PMHS**) interactions.

Consequently, the aims of this study were: (i) to investigate the effect of prenatal dam health status during gestation on the F1 disease prevalence for DIAR and PNEU, and on production levels in the F1 offspring, (ii) to infer genetic (co)variance components for DIAR and PNEU in dependency of the PMHS and (iii) to apply RRM for DIAR and PNEU by considering the average prevalence for the PMHS within herd‐calving‐years as continuous “environmental descriptor.”

## 2 Materials and Methods

### 2.1 Phenotypes

The starting point for the analyses was a disease dataset of 20,045 Holstein Friesian (**HF**) female calves, being offspring from second parity cows. Previous data editing only considered the exclusion of twins (2.8% of all births), because of the twin calving difficulties with possible effects on the ongoing health status (Mahmoud et al. 2017). In a next step, the calf health dataset was merged with the health and production trait datasets of the respective 20,045 dams. Calves and cows were kept in 41 large‐scale co‐operator herds from the two German federal states Mecklenburg‐Western Pomerania and Berlin‐Brandenburg. Birth years of the cows (= dam generation 0, **F0**) spanned the period from 2003 to 2013. The calves (= generation 1, **F1**) were born in the years 2008 to 2016. Due to the fact that all F1 calves were from their dams' 2^nd^ parity, all dam (maternal) diseases were recorded during the first lactation and dry period. The mean age at second calving of the dams was 38.53 months with a SD of 2.88 months. Cow and calf diseases were diagnosed by veterinarians or trained herd managers according to the “central key for health data recording” from the official “International Committee for Animal Recording” nomenclature (Stock et al. 2013). The diseases were binary recorded (0 = healthy; 1 = diseased) in both generations F0 cows and F1 calves within defined time periods. The F0 cows were phenotyped for mastitis (**MAST**) and for claw disorders (**CD**) during whole gestation. According to the diagnosis key, all cows with an entry for at least one inflammation of the udder were classified as MAST‐diseased cows. All cows with at least one diagnosis for dermatitis digitalis or dermatitis interdigitalis were classified as CD‐diseased cows. The F1 calves were phenotyped for DIAR until 30 days post‐partum, and for PNEU until 180 days post‐partum. Only calves who had the opportunity to reach 30 and 180 days of life for DIAR and PNEU, respectively, were included in the analysis. Hence, the numbers of observations slightly differed for both calve diseases (Table 1). The defined periods for both calf diseases are in agreement with data preparations by Ames (1997) and Berber et al. (2021). The diagnosis DIAR comprised all types of a diarrhoea syndrome. The calf disease PNEU included inflammation symptoms of the bronchi and the lungs. At least one entry for the respective calf disease in the observation period implied the score = 1 = diseased, otherwise, the score = 0 = healthy, was assigned. The combination of F0 dam and F1 calf diseases implied four different association groups: (1) MAST diagnosis (0 = healthy; 1 = diseased) of F0 cows and DIAR diagnosis (0 = healthy; 1 = diseased) of F1 calves, (2) MAST diagnosis of F0 cows and PNEU diagnosis of F1 calves, (3) CD diagnosis of F0 cows and DIAR diagnosis of F1 calves, and (4) CD diagnosis of F0 cows and PNEU diagnosis of F1 calves. The number of observations and the disease prevalence for all calf‐cow disease status combinations are given in Table 1. The final datasets used for ongoing association analyses and genetic studies slightly differed from the original dataset including 20,045 calf‐cow pairs due to strict data editing criteria related to possible inconsistencies, e.g., possible typos for a specific diagnosis or comments related to a diagnosis indicating that the respective diagnosis was not 100% clear.

**TABLE 1:** Overview of the number of observations and prevalences for F0 dam disease status in first lactation and for corresponding F1 calf disease status.

F0 dam disease	F0 dam status	F1 calf disease	F1 calf status	No. of observations	Prevalence (in %) F0 dam disease	Prevalence (in %) F1 calf disease
Mastitis	Healthy	Diarrhoea	Healthy	14,625	—	—
	Healthy	Diarrhoea	Diseased	3059	—	17.30
	Diseased	Diarrhoea	Healthy	1627	10.01	—
	Diseased	Diarrhoea	Diseased	527	14.70	24.47
Total				19,838	10.86	18.08
Mastitis	Healthy	Pneumonia	Healthy	12,919	—	—
	Healthy	Pneumonia	Diseased	4551	—	26.05
	Diseased	Pneumonia	Healthy	1410	9.84	—
	Diseased	Pneumonia	Diseased	693	13.22	32.95
Total				19,573	10.74	26.79
Claw disorders	Healthy	Diarrhoea	Healthy	14,522	—	—
	Healthy	Diarrhoea	Diseased	3261	—	18.34
	Diseased	Diarrhoea	Healthy	1730	10.64	—
	Diseased	Diarrhoea	Diseased	325	9.06	15.82
Total				19,838	10.36	18.08
Claw disorders	Healthy	Pneumonia	Healthy	12,858	—	—
	Healthy	Pneumonia	Diseased	4680	—	26.68
	Diseased	Pneumonia	Healthy	1471	10.27	—
	Diseased	Pneumonia	Diseased	564	10.76	27.71
Total				19,537	10.40	26.79

Records for 305‐d lactation milk yield (**MY**), protein yield (**PY**) and fat yield (**FY**) in first lactation were available from 10,129 F1 cows (= offspring of the F0 dams). The dataset reduction from F1 calves with disease diagnoses to F1 cows with completed 305‐d lactations is due to sales of pregnant heifers and first parity cows, and due to involuntary cullings during first lactation. The age at first calving of F1 cows ranged from 20 to 38 months (mean: 25.38 months; SD: 2.30 months). The combination of F0 dam diseases and F1 cow 305‐d lactation traits implied two further association groups: (5) MAST (0 = healthy; 1 = diseased) of F0 cows and MY, FY and PY of F1 cows, and (6) CD (0 = healthy; 1 = diseased) of F0 cows and MY, FY and PY of F1 cows. Descriptive statistics for MY, PY and FY with regard to dam groupings are given in Table 2.

**TABLE 2:** 305‐d production trait records in first lactation of F1 cows in relation to the disease status of their F0 dams for mastitis and claw disorders.

F0 dam disease	F0 dam disease status	F1 cow trait	No. of obs.	Mean	SD	Min	Max
Mastitis	Healthy	MY	9130	8954.16	1625.21	2285.00	15663.00
	Diseased		999	8858.76	1667.25	2734.00	14823.00
	Healthy	FY	9130	343.08	56.56	97.00	572.00
	Diseased		999	338.49	58.46	117.00	513.00
	Healthy	PY	9130	298.46	49.33	81.00	491.00
	Diseased		999	294.98	50.46	103.00	447.00
Claw disorders	Healthy	MY	9194	8937.85	1634.85	2285.00	15663.00
	Diseased		935	9012.65	1576.00	3649.00	14051.00
	Healthy	FY	9194	342.31	56.77	97.00	572.00
	Diseased		935	345.68	56.60	141.00	529.00
	Healthy	PY	9194	297.88	49.61	81.00	491.00
	Diseased		935	300.46	47.85	130.00	445.00

Abbreviations: FY, fat yield in kg; MY, milk yield in kg; PY, protein yield in kg.

The PHMS calculations within herd‐calving‐years considered disease records from all F1 dams (the 20,045 cows) and their respective herd contemporaries (additional 16,193 cows). Due to the small number of observations for herd‐calving‐year prevalences larger than 0.30 for MAST and CD, herd‐calving‐years with a prevalence larger than 0.30 were excluded from the RRM analyses. Specifically, only 5% of all calf records were allocated to extreme herd‐calving‐years with a prevalence for MAST larger than 30%. Similarly, herd‐calving‐years with a prevalence for CD larger than 0.30 only comprised 8% of all calf records. Hence, the continuous “PMHS herd‐calving‐year descriptor” in all RRM analyses considered a prevalence range from 0% to 30%. Consequently, due to the different dataset preparation strategies for the bivariate models and for the RRM, parameter estimates are not fully comparable.

### 2.2 Pedigree and Genotypes

The pedigree file included in total 84,088 animals. The ancestors of the phenotyped F0 cows and F1 calves could be traced back to at least three generations. Genotype data were provided by the national genetic evaluation centre vit Verden. In this regard, we used the imputed SNP data including 45,613 SNPs from 9762 cattle. 6561 cattle were genotyped using the *Illumina BovineSNP50 v2 BeadChip* (Illumina Inc.), and the remaining 3201 cattle using the *Eurogenomics 10 K low‐density chip* (EuroGenomics Cooperative U.A., Amsterdam, The Netherlands). The imputation algorithm as applied by vit Verden is described by Segelke et al. (2012). For the imputed SNP dataset, we performed further quality controls using the program preGSf90 from the BLUPf90 package (Misztal et al. 2014). In this regard, the following criteria were applied: consideration of SNPs located on autosomes; exclusion of SNPs with a call rate lower than 0.95, a minor allele frequency lower than 0.05 and a significant deviation from Hardy–Weinberg equilibrium; exclusion of genotyped cattle with a call rate less than 0.95 and with a genomic relatedness larger than 0.95. After quality control, the genotype dataset included 41,147 SNPs from 9762 cattle, i.e., 2425 genotyped F0 dams, 2204 genotyped F1 females and 5133 genotyped related animals.

### 2.3 Statistical models

#### 2.3.1 Phenotypic Effects of the Prenatal Maternal Health Status on Offspring Diseases and Production

A generalised linear mixed model with a logit link function as implemented in the GLIMMIX procedure of SAS (SAS OnDemand for Academics, SAS Institute; Cary, NC, USA) was applied to estimate the effect of PMHS during gestation on F1 calf diseases. The applied statistical model 1 was:

(1) $$\begin{array}{c} \text{logit}\left(\pi_{\text{rstuvw}}\right)=\log\;\left[\pi_{\text{rstuvw}}/\left(1-\pi_{\text{rstuvw}}\right)\right]\\ \;=\varphi +{\text{Herd}}_{r}+{\mathrm{BY}}_{s}+{\mathrm{BM}}_{t}+\mathrm{CA}\_D_{u}+{\text{PMHS}}_{v}+{\mathrm{GL}}_{w} \end{array}$$

where $\pi_{\text{rstuvw}}$ = probability for a calf to get diseased (DIAR or PNEU; 0 = healthy, 1 = diseased); *φ* = overall mean effect; Herd_*r*_ = fixed effect of herd (*r* = 1–41); BY_*s*_ = fixed effect of calf birth year (*s* = 2008–2016); BM_*t*_ = fixed effect of calf birth month (*t* = 1 to 12); CA_D_*u*_ = fixed effect of calving age of the dam (*u* = ≤ 34, 35, …, 45, ≥ 46 months); PMHS_*v*_ = fixed effect of PMHS (MAST or CD, 0 = healthy, 1 = diseased); GL_*w*_ = linear regression on gestation length of the dam (*w* = 265–295 days). We considered the gestation length of the dam in the model, because Vieira‐Neto et al. (2017) indicated significant effects of gestation length on both offspring diseases DIAR and PNEU.

A fixed effects model was used to determine the effects of F0 cow PMHS during gestation on F1 cow 305‐d lactation traits. The defined statistical model 2 was:

(2) $$y_{\text{ijklmn}}=\mu +{\text{Herd}}_{i}+{\mathrm{CY}}_{j}+{\mathrm{CM}}_{k}+{\mathrm{CA}}_{l}+{\text{PMHS}}_{m}+{\mathrm{GL}}_{n}+e_{\text{ijklmn}}$$

where y_*ijklmn*_ = observations for 305‐d milk production traits (MY, PY and FY) of F1 cows; *μ* = overall mean effect; Herd_*i*_ = fixed effect of herd (*i* = 1–41); CY_*j*_ = fixed effect of cow calving year (*j* = 2009–2016); CM_*k*_ = fixed effect of cow calving month (*k* = 1–12); CA_*l*_ = fixed effect of calving age of the cow (*l* = ≤ 22, 23, …, 30, ≥ 31 months); PMHS_*m*_ = fixed effect of PMHS (MAST or CD, 0 = healthy, 1 = diseased); GL_*n*_ = linear regression on days in milk (*n* = 201 to 305 days); and *e*_*ijklmn*_ = random residual effect.

Significances of fixed effects were determined via F‐tests (type III tests of fixed effects). We estimated least squares means (**LSM**) and pairwise differences of LSM for calf diseases (DIAR and PNEU) and for 305‐d milk production traits of F1 cows with respect to MAST and CD groups of F0 dams. Thus, LSM comparisons considered the following groupings: DIAR‐MAST_healthy_ with DIAR‐MAST_diseased_, DIAR‐CD_healthy_ with DIAR‐CD_diseased_, PNEU‐MAST_healthy_ with PNEU‐MAST_diseased_, PNEU‐CD_healthy_ with PNEU‐CD_diseased_, and in addition, the comparisons for F1 production traits from healthy and diseased dam groups. The significance value for the F‐tests and for pairwise differences of LSM was set at *p* ≤ 0.05.

### 2.4 Direct Genetic and Maternal Genetic Parameters of Calf Diseases in Dependency of the Prenatal Maternal Health Status

Bivariate animal models were applied in BLUPf90 (Misztal et al. 2014) to estimate direct and maternal genetic (co)variance components for DIAR and PNEU with respect to the PMHS. In this regard, same calf traits from different dam health groups were defined as different traits, i.e., DIAR‐MAST_healthy_ and DIAR‐MAST_diseased_, DIAR‐CD_healthy_ and DIAR‐CD_diseased_, PNEU‐MAST_healthy_ and PNEU‐MASTd_iseased_, and PNEU‐CD_healthy_ and PNEU‐CD_diseased_. The trait definition strategy implied four consecutive bivariate runs according to the combinations of calf and cow diseases. The respective genetic‐statistical bivariate model 3 in matrix notation was:

(3) $$\left[\begin{array}{c} y_{1}\\ y_{2} \end{array}\right]=\left[\begin{array}{ccccccc} X_{1}b_{1} & + & Z_{1}d_{1} & + & W_{1}m_{1} & + & e_{1}\\ X_{2}b_{2} & + & Z_{2}d & + & W_{2}m_{2} & + & e_{2} \end{array}\right]$$

where $y_{1}$ = vector of observations for DIAR and PNEU from healthy dams; $y_{2}$ = vector of observations for DIAR and PNEU from diseased dams; b = vector for fixed effects as specified in model 1; ***d*** = vector for direct additive genetic effects, m = vector for maternal genetic effect; e = vector for random residual effects; and X,Z and **W** = design matrices for b,d and m, respectively. The (co)variance structure for random effects was:

$$\mathrm{var}\;\left[\begin{array}{c} d_{1}\\ d_{2}\\ m_{1}\\ m_{2}\\ e_{1}\\ e_{2} \end{array}\right]=\left[\begin{array}{cccccc} H\sigma_{d_{1}}^{2} & H\sigma_{d_{1}d_{2}} & H\sigma_{d_{1}m_{1}} & H\sigma_{d_{1}m_{2}} & 0 & 0\\ H\sigma_{d_{1}d_{2}} & H\sigma_{d_{2}}^{2} & H\sigma_{d_{2}m_{1}} & H\sigma_{d_{2}m_{2}} & 0 & 0\\ H\sigma_{d_{1}m_{1}} & H\sigma_{d_{2}m_{1}} & H\sigma_{m_{1}}^{2} & H\sigma_{m_{1}m_{2}} & 0 & 0\\ H\sigma_{d_{1}m_{2}} & H\sigma_{d_{2}m_{2}} & H\sigma_{m_{1}m_{2}} & H\sigma_{m_{2}}^{2} & 0 & 0\\ 0 & 0 & 0 & 0 & I_{n_{1}}\sigma_{e_{1}}^{2} & 0\\ 0 & 0 & 0 & 0 & 0 & I_{n_{2}}\sigma_{e_{2}}^{2} \end{array}\right]$$

where $\sigma_{d_{1}}^{2}$ and $\sigma_{d_{2}}^{2}$ = the direct genetic variances for the same calf disease from healthy and diseased dams, respectively; $\sigma_{m_{1}}^{2}$ and $\sigma_{m_{2}}^{2}$ = the maternal genetic variances for the same calf disease from healthy and diseased dams, respectively; $\sigma_{d_{1}m_{1}}$ and $\sigma_{d_{2}m_{2}}$ = covariances between direct and maternal genetic effects for the same calf disease from healthy and diseased dams, respectively; $\sigma_{d_{1}d_{2}}$ = covariance between direct genetic effects for the same calf disease from healthy and diseased dams; $\sigma_{m_{1}m_{2}}$ = covariance between maternal genetic effects for the same calf disease from healthy and diseased dams; $\sigma_{d_{1}m_{2}}$ and $\sigma_{d_{2}m_{1}}$ = covariances between direct genetic effects for the same calf disease from healthy dams with maternal genetic effects from diseased dams, and vice versa, respectively; $\sigma_{e_{1}}^{2}$ and $\sigma_{e_{2}}^{2}$ = residual variances for the same calf disease from healthy and diseased dams, respectively; **H** = the combined genetic relationship matrix, which was computed by blending the pedigree relationship matrix **A** and the weighted genomic relationship matrix (**G**_**w**_) (Legarra, Aguilar, and Misztal 2009) with **G**_**w**_ = (0.95 × **G** + 0.05 × **A**_**22**_), where **G** represents the genomic relationship matrix (VanRaden 2008), and **A**_**22**_ was the submatrix of the pedigree‐based relationship matrix for genotyped animals; $I_{n_{1}}$ and $I_{n_{2}}$ = identity matrices for calves from healthy (*n*_1_) and diseased dams (*n*_2_), respectively.

For inferring genetic parameters of DIAR and PNEU in dependency of the prenatal herd health status, a single‐step RRM including the herd‐calving‐year prevalence for MAST and for CD as continuous “environmental descriptor” was applied. In matrix notation, the respective statistical model 4 was defined as follows:

(4) y=Xb+Zu+Wm+e

where **y** = vector of observations for DIAR or for PNEU; **b** = vector of fixed effects including herd, calf birth year, calf birth month, gestation length of the dam and herd‐calving‐year prevalence for MAST or CD nested within the herd effect, **u** = vector of random regression coefficients for slope and intercept of direct genetic effects on herd‐calving‐year prevalence (modelled as linear regression) for MAST or CD; **m** = vector of random maternal genetic effects; and **e** = vector of random residual effects; and **X**, **Z** and **W** = the incidence matrices for **b**, **u** and **m**, respectively. The modelling approach allows the estimation of additive‐genetic variances and direct heritabilities on the continuous herd‐calving‐year prevalence scale. Alterations of maternal heritabilities for DIAR and PNEU are only due to variations in proportions of maternal variances in relation to the total variances.

The variance–covariance structure for the random effects was assumed as:

$$\mathrm{var}\left[\begin{array}{c} d\\ m\\ e \end{array}\right]=\left(\begin{array}{ccc} G_{a}⨂H & g_{\mathrm{dm}}⨂H & 0\\ g_{\mathrm{dm}}^{\prime }⨂H & H\sigma_{m}^{2} & 0\\ 0 & 0 & \sigma_{e}^{2}I_{n} \end{array}\right)$$

where **G**_**a**_ = 2 × 2 additive genetic (co)variance matrix for random regression coefficients of direct genetic effects; $g_{\mathrm{dm}}$= vector of additive genetic covariances between random regression coefficients for direct genetic effects and maternal genetic effects; $\sigma_{m}^{2}$ = variance for maternal genetic effects; **H** = the combined genetic relationship matrix as explained above; $\sigma_{e}^{2}$ = random residual variance; ***I***_***n***_ = identity matrix for *n* observations, and ⨂ = Kronecker product.

## 3 Results

### 3.1 Phenotypic Effects of the Prenatal Maternal Health Status on Offspring Diseases and Production Traits

**FIGURE 1:**
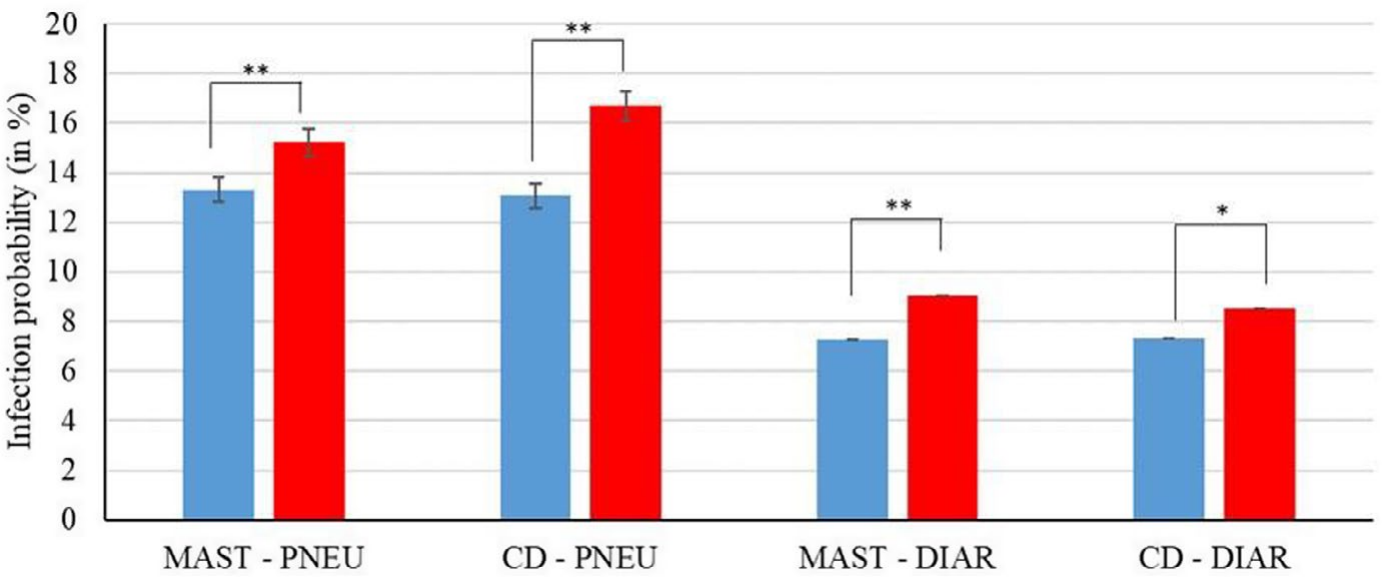
Least‐squares means for the probability to get diseased with diarrhoea (DIAR) or pneumonia (PNEU) of F1 calves from diseased (= red bars) and healthy (= blue bars) dam groups for mastitis (MAST) and claw disorders (CD) (** = significant differences at *p* < 0.01; * = significant differences at *p* ≤ 0.05). [Colour figure can be viewed at wileyonlinelibrary.com]

Results from the overall *F*‐test indicate that a prenatal diagnosis for MAST and CD had a significant unfavourable effect on DIAR (*p* = 0.001 and *p* = 0.044, respectively), and on PNEU (*p* = 0.007 and *p* = 0.0001, respectively). The LSM for the probabilities of a DIAR disease and for a PNEU disease were significantly higher when a dam had a MAST or CD diagnosis during gestation compared to the healthy dam group (Figure 1). The pairwise difference between the LSM of DIAR‐MAST_diseased_ and DIAR‐MAST_healthy_ groups was 1.74%, and 1.91% between PNEU‐MAST_diseased_ and PNEU‐MAST_healthy_ groups (Figure 1). Similarly, the pairwise difference between LSM of DIAR‐CD_diseased_ and DIAR‐CD_healthy_ groups was 1.21%, and 3.61% between the PNEU‐CD_diseased_ and PNEU‐CD_healthy_ group (Figure 1).

In ongoing association analyses, we considered both maternal diseases MAST and CD simultaneously as a fixed effect for the overall infection status of the dam, i.e., the dam was diseased by either MAST or by CD during gestation. However, pairwise differences between LSM for DIAR and PNEU remained in the same range from 1.2% to 3.7%, indicating a lower disease prevalence of calves from healthy dams (results not shown).

**FIGURE 2:**
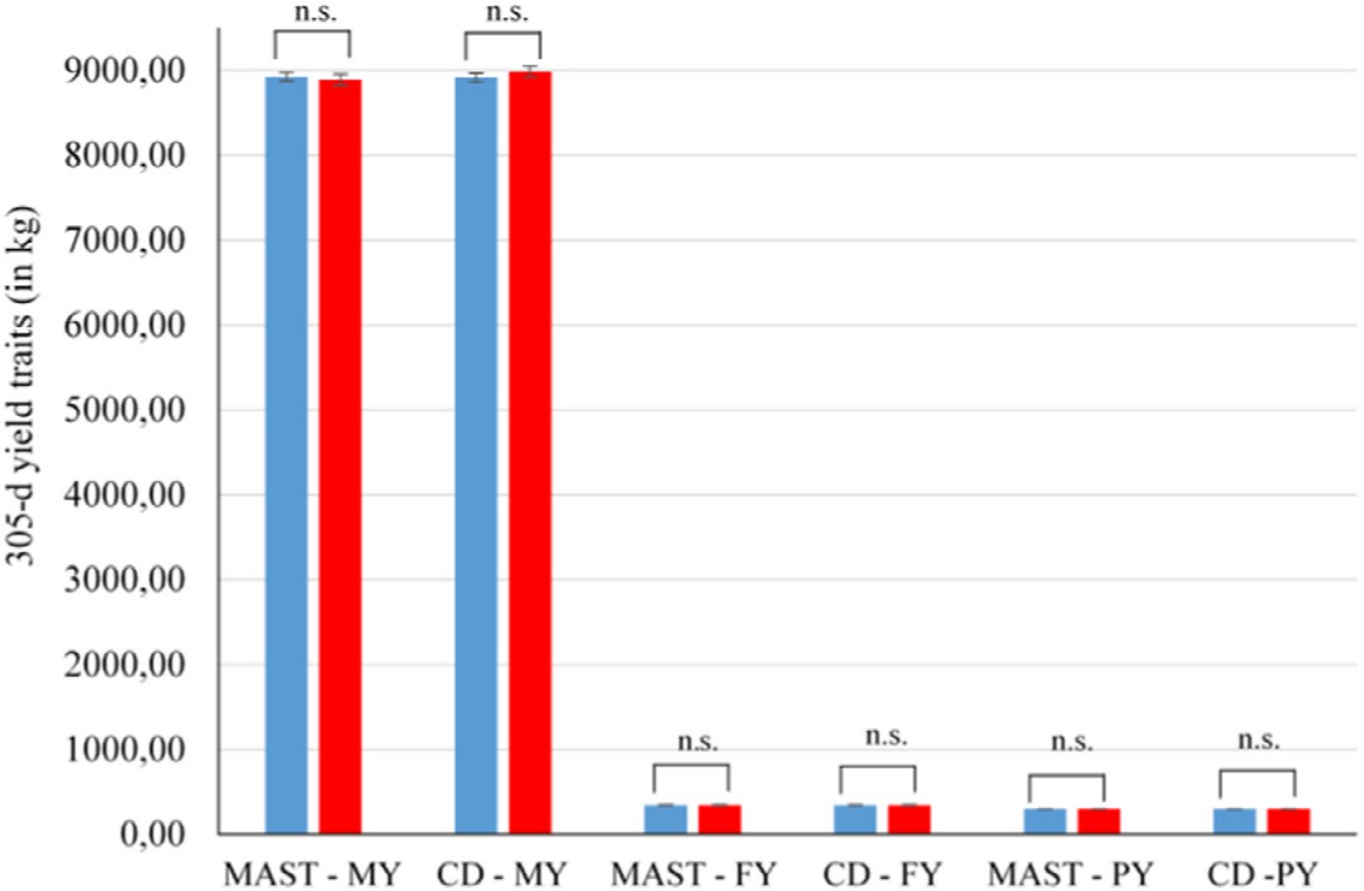
Least‐squares means for 305‐d lactation records for milk yield (MY), fat yield (FY) and protein yield (PY) of first parity F1 cows from diseased (= red bars) and healthy (= blue bars) dam groups for mastitis (MAST) and claw disorders (CD) (n.s. = non‐significant differences with *p* > 0.05). [Colour figure can be viewed at wileyonlinelibrary.com]

With regard to F1 production traits, the fixed effects of MAST or CD grouping in F0 cows on the 305‐d lactation traits MY, FY and PY in the first lactation of F1 offspring were non‐significant (*p* > 0.05; results from overall *F*‐test). The respective LSM for MY, FY and PY with regard to the maternal health status for MAST and CD are displayed in Figure 2.

### 3.2 Genetic Parameters for Calf DIAR and PNEU in Dependency of the Prenatal maternal Health Status

The direct and maternal heritabilities from the bivariate analyses for DIAR and PNEU of F1 calves in MAST‐diseased versus non‐diseased F0 dam groups, and in CD‐diseased versus non‐diseased F0 dam groups, are given in Table 3. The direct heritability for DIAR was 0.11 for the MAST_healthy_ group, and very similar to 0.13 for the MAST_diseased_ group. With regard to CD dam grouping, stronger differences between DIAR heritabilities were observed. The DIAR heritability was 0.11 in the CD_healthy_ group, and 0.04 in the CD_diseased_ group. For PNEU, the direct heritability in the four dam groups ranged from 0.13 (dams with a MAST diagnosis) to 0.22 (dams with a CD diagnosis). The PNEU heritability was significantly smaller (0.14) in the CD_healthy_ dam group than in the CD_diseased_ dam group (0.22). The heritability difference was due to the larger genetic variance for PNEU within the CD‐diseased dam group compared to the respective healthy dam group.

**TABLE 3:** Estimated heritabilities^a^ and genetic correlations^a^ for the calf diseases within and between the F0 dam disease groups for mastitis and claw disorders. Significant differences for heritabilities^b^ and genetic correlations^c^ are indicated as footnotes.

F0 dam disease	F1 calf disease	Healthy F0 dam group			Diseased F0 dam group			Healthy and diseased F0 dam	
		*h* ^2^_*d*_	*h* ^2^_*m*_	*r*_*dm*_	*h* ^2^_*d*_	*h* ^2^_*m*_	*r*_*dm*_	*r*_*d*_	*r*_*m*_
Mastitis	Diarrhoea	0.11 ± 0.01	0.10 ± 0.01	−0.90 ± 0.03	0.13 ± 0.03	0.09 ± 0.02	−0.75 ± 0.08	0.80 ± 0.06	0.80 ± 0.07
	Pneumonia	0.14 ± 0.02	0.08 ± 0.02	−0.87 ± 0.04	0.13 ± 0.04	0.05 ± 0.01	−0.88 ± 0.08	0.88 ± 0.17	0.87 ± 0.12
Claw disorders	Diarrhoea	0.11 ± 0.01	0.10 ± 0.01	−0.89 ± 0.02	0.04 ± 0.01	0.04 ± 0.05	−0.43 ± 0.08	0.74 ± 0.04	0.61 ± 0.06
	Pneumonia	0.14 ± 0.01	0.09 ± 0.01	−0.87 ± 0.02	0.22 ± 0.11	0.11 ± 0.02	−0.52 ± 0.08	0.64 ± 0.06	0.35 ± 0.09

^a^ *h* ^2^_*d*_ = direct heritability; *h* ^2^_*m*_ = maternal heritability; *r*_*dm*_ = correlation between direct and maternal genetic effects; *r*_*d*_ = direct genetic correlations between the cow groups; *r*_*m*_ = Maternal genetic correlations between the cow groups.

^b^ The direct heritabilities and the maternal heritabilities for diarrhoea and pneumonia with regard to F0 mastitis dam grouping did not differ significantly (*p* > 0.05). The direct heritabilities and the maternal heritabilities for diarrhoea with regard to F0 claw disorder dam grouping differed significantly (*p* ≤ 0.05). The direct heritabilities for pneumonia with regard to F0 claw disorder dam grouping differed significantly (*p* ≤ 0.05). The maternal heritabilities for pneumonia with regard to F0 claw disorder dam grouping did not differ significantly (*p* > 0.05).

^c^ The following genetic correlations significantly differed (*p* ≤ 0.05) from 0.80: the maternal genetic correlation for diarrhoea with regard to F0 claw disorder dam grouping, the direct genetic correlation for pneumonia with regard to F0 claw disorder dam grouping, the maternal genetic correlation for pneumonia with regard to F0 claw disorder dam grouping.

The maternal heritabilities (Table 3) were quite similar for DIAR and PNEU across PMHS groups and ranged from 0.04 to 0.11. The maximal maternal heritability difference was 0.06, i.e., for DIAR and the dam grouping according to CD. With regard to PNEU and prenatal dam CD, the direct heritabilities differed significantly. The respective minor difference of 0.02 for the maternal heritability for PNEU between the CD dam groups indicates stronger time‐lagged stressor effects on direct genetic than on maternal genetic effects.

Table 3 additionally presents correlations for direct and maternal genetic effects, considering DIAR and PNEU from different F0 dam groups as different traits. The direct genetic correlations between the same calf diseases from different dam groups were in the range from 0.64 to 0.88, and from 0.35 to 0.87 for the maternal genetic correlations. Genetic correlations between direct genetic and maternal genetic effects for DIAR and PNEU in MAST_diseased_ and in MAST_healthy_ dam groups, and between direct genetic and maternal genetic effects in CD_diseased_ and CD_healthy_ dam groups, were throughout negative (Table 3). For the DIAR and MAST dam grouping, the correlations were − 0.90 (in the healthy dam group) and −0.75 (in the diseased dam group), and very similar for PNEU in both MAST dam groups with −0.87 and −0.88, respectively. The direct‐maternal genetic correlations based on the prenatal CD dam health status displayed larger differences between the healthy and diseased dam groups with −0.89 and −0.43 for DIAR, and −0.87 and −0.52 for PNEU, respectively.

### 3.3 Genetic parameters for calf DIAR and PNEU on the continuous prenatal herd‐calving‐year prevalence scale

**FIGURE 3:**
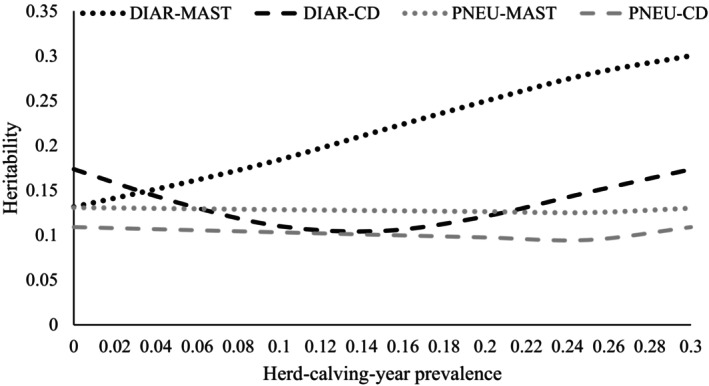
Direct heritabilities for the calf diseases diarrhoea (DIAR) and pneumonia (PNEU) in dependency of the average herd‐calving‐year prevalence for mastitis (MAST) and claw disorders (CD) considering the whole gestation period of the dam. Standard errors of heritabilities along the herd‐calving‐year scale were in the range from 0.002 to 0.02.

Direct heritabilities from the RRM for DIAR and PNEU along the continuous herd‐calving‐year prevalence scale are presented in Figure 3. The direct heritabilities for PNEU were quite stable along the prevalence levels for MAST and for CD. Larger fluctuations for direct heritabilities were observed for DIAR in dependency of the prevalence herd‐calving‐year levels for CD and especially for MAST. For “DIAR‐CD,” direct heritabilities increased at the extreme ends of the herd‐calving‐year scale. The direct heritabilities for DIAR were 0.17 at the herd‐calving‐year CD prevalence of 0% and of 30%, and smallest (0.10) at the intermediate prevalence of 15%. A quite strong direct heritability increase was observed for DIAR in dependency of the herd‐calving‐year prevalence for MAST, i.e., from 0.13 (at a MAST prevalence of 0%) to 0.30 (at a MAST prevalence of 30%). The strong increase of the direct heritabilities was due to the increase of the additive‐genetic variances with increasing herd‐calving‐year prevalence. In all RRM runs, SE of direct heritabilities were quite small in the range from 0.002 to 0.02.

In analogy with results from the bivariate modelling approach, the maternal heritabilities were smaller than the respective direct heritabilities for PNEU and DIAR. Due to the modelling strategy considering only random regressions for the direct‐genetic effect, the maternal heritabilities were quite stable along the continuous herd‐calving‐year prevalence scale, i.e., 0.06 for “PNEU‐MAST,” in the range from 0.04 to 0.05 for “PNEU‐CD,” and in the range and from 0.05 to 0.07 for “DIAR‐CD.” The maternal heritability for DIAR in dependency of the herd‐calving‐year prevalence for MAST indicated a slight decline from 0.07 (at a MAST prevalence of 0%) to 0.03 (at a MAST prevalence of 30%) due to the increasing phenotypic variance (i.e., an effect of the increasing direct genetic variance) along the herd‐calving‐year scale.

**FIGURE 4:**
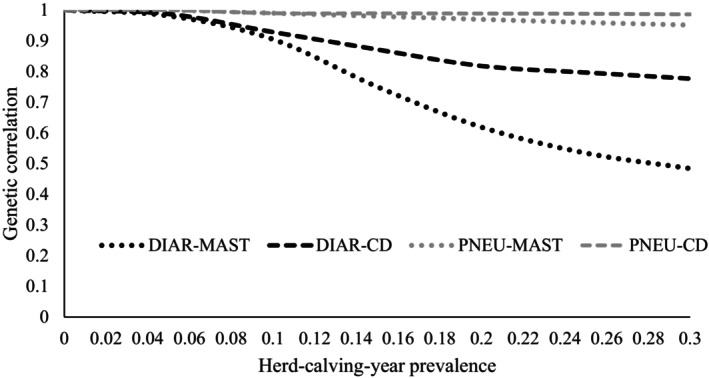
Genetic correlations between the same calf trait (DIAR or PNEU) at a herd‐calving‐year prevalence of 0% (for MAST or for CD considering the whole gestation period of the dam) with DIAR or PNEU at the remaining levels for the MAST prevalence or the CD prevalence. Standard errors of correlations along the herd‐calving‐year scale were in the range from 0.002 to 0.014.

The genetic correlations between the same calf disease (PNEU or DIAR) at a herd‐calving‐year prevalence of 0% for MAST or for CD with remaining levels for the herd‐calving‐year prevalence are depicted in Figure 4. The chosen random regression modelling strategy with the herd‐calving‐year prevalence as continuous environmental descriptor on the x‐axis enables the estimation of genetic correlations between the same calf disease for all other prevalence combinations, but for simplification, we focused on the “0% prevalence for MAST or for CD” with all remaining prevalences. Nevertheless, the general curve pattern, i.e., highest genetic correlations between the same trait for neighbouring MAST or CD prevalence levels, was observed for all other combinations. For the calf‐cow trait combinations “PNEU‐CD” and “PNEU‐MAST,” the correlations were throughout larger than 0.90. In the present study, an obvious decline in genetic correlations across the herd‐calving‐year prevalence scale was observed for DIAR in dependency of prenatal MAST (Figure 4). The genetic correlation estimates decreased from 0.98 (at 0% CD prevalence) to 0.48 (at 30% CD prevalence).

The direct‐maternal genetic correlations for same calf traits along the continuous herd‐calving‐year prevalence scale reflect the antagonistic estimates from the bivariate model. Correlations were quite stable for “PNEU‐MAST” in the range from −0.82 to −0.78, and for “PNEU‐CD” in the range from −0.85 to −0.82. Again, larger estimate fluctuations were observed for DIAR, with a direct‐maternal genetic correlation of −0.76 at a MAST prevalence of 0% and of −0.56 at a MAST prevalence of 30%, and in a range from −0.68 to −0.82 for “DIAR‐CD.”

## 4 Discussion

### 4.1 Phenotypic Effects of the Prenatal Maternal Health Status on Offspring Diseases and Production Traits

An increased risk for a DIAR and a PNEU infection in offspring of diseased dams indicates prenatal immune activation induced by bacterial pathogens for MAST or CD during gestation. A prenatal immune activation in response to mastitis was described by Hansen, Soto, and Natzke (2004). For the dam disease grouping, we explicitly focused on the bacterial‐induced diseases MAST and CD, because MAST and CD are the most prevalent diseases in dairy cows (Thompson‐Crispi et al. 2014; Palmer and O'Connell 2015). As outlined by Moe et al. (2010) and Schukken et al. (2011), a MAST or a CD infection was associated with a local and a systemic host immune activation, causing alterations in circulating antibody levels in serum. In dairy cows, a systemic activation of immune and inflammatory responses during MAST increased the risk for embryonic losses in the early stage of implantation (Hansen, Soto, and Natzke 2004). In contrast to our findings, Carvalho et al. (2020) observed that daughters of dams diseased during gestation had a lower infection risk for digestive disorders compared to daughters from healthy dams. These intergenerational effects might be due to genetic and non‐genetic inherited factors (e.g., epigenetics, microbiota) including a heritable component, as well as due to indirect environmental in utero effects (David et al. 2019). The contribution of bacterial and viral infections on epigenetic modifications, i.e., on DNA methylations and histone modifications, has been described in several species (Vanselow et al. 2006; Weber‐Stadlbauer et al. 2017). In HF cows, Vanselow et al. (2006) showed that *Escherichia coli* and *Staphylococcus aureus* infections in the mammary gland were related with CpG methylation pattern of milk genes. However, the systemic effects of pathogenic infections on the epigenome and the possible transmission of epigenome modifications across generations, are unknown.

In addition to maternal health disorders, heat stress during gestation is another stressor with proven time‐lagged effects on disease incidences and performances in HF cows (Yin, Halli, and König 2022). One explanation of detrimental time‐lagged stress effects addressed immune system mechanisms, especially impaired passive and cell‐mediated immune functions in calves (Tao et al. 2012; Monteiro et al. 2014). Based on data from large‐scale HF contract herds, Kipp et al. (2021b) and Yin, Halli, and König (2022) associated stress effects during the last third of gestation with increased disease occurrences and lower productivity in offspring. The period comprising the last third of gestation mainly determined fetal growth and proliferation of immune cells in bovine foetus (Higgins, Stack, and Richardson 1983). González‐Recio, Ugarte, and Bach (2012) focused on the period of embryogenesis and indicated impaired cow longevity and milk yield due to maternal udder infections. With regard to the maternal diseases MAST and CD in the present study, we did not differentiate between different periods of gestation. However, there was a tendency (results are not shown) for strongest detrimental effects on offspring DIAR and PNEU due to MAST or CD diagnoses during the third trimester of gestation. A MAST or CD disease diagnosis within the third trimester of gestation was associated with smaller colostrum quantity (Maunsell et al. 1998), and might increase the risk for impaired colostrum quality (e.g., immunoglobulin G [IgG] content). In consequence, indirect effects on calf health can be postulated, because inadequate intake of colostrum post‐partum increased the susceptibility to bacterial infections, leading to DIAR and PNEU in dairy calves (Donovan et al. 1998; Arsenopoulos, Theodoridis, and Papadopoulos 2017). Moreover, as shown in small ruminants, the bacterial milk composition altered with respect to the udder health status, and was related with disease susceptibility in offspring (Toquet, Gómez‐Martín, and Bataller 2021). In livestock, David et al. (2019) highlighted the pronounced genetic component of the microbiota, meaning that intergenerational transmission inheritance of disease susceptibility can be explained by the microbiota component as well.

In the present study, the effect of the PMHS was non‐significant for 305‐d production traits in offspring. With regard to prenatal maternal CD, we observed even minimal higher MY of F1 cows allocated to the diseased dam group. This is in agreement with results by Kipp et al. (2021a), indicating time‐lagged detrimental heat stress effects from late gestation on functional traits, but not on production traits. In such context, there is a lack of studies investigating trait‐specific physiological or genetic mechanisms. However, we assume an effect due to selection. For the calf diseases, the datasets comprised records from almost 20,000 calves. For later 305‐d cow production traits, records from 10,129 F1 cows were available. As pointed out by Shabalina, Yin, and König (2020), the level of test‐day milk yield in early lactation is a major selection criterion in German Holstein herds. Consequently, low‐yielding cows are leaving the herds in the first third of the first lactation before completing a 305‐d lactation record.

### 4.2 Genetic Parameters for Calf DIAR and PNEU in Dependency of the Prenatal Maternal Health Status

For all created cow groups, direct heritabilities for DIAR and PNEU reflect the heritability range (0.10–0.17) as reported by Haagen et al. (2021) and Yin, Halli, and König (2022) for single‐trait model applications in HF populations. The largest direct heritability of 0.22 for PNEU in the CD_diseased_ dam group indicates a more pronounced genetic differentiation of health traits under challenging PMHS conditions. Alterations of heritabilities for disease indicator traits were mostly studied from a prompt stressor impact instead of modelling time‐lagged stress effects. In this regard, Windig et al. (2006) studied the effect of the cow herd production environment on genetic parameter estimates for somatic cell score and identified pronounced heritability alterations due to production intensity. In HF cattle, Shi et al. (2021) reported slightly larger direct heritabilities for cortisol under heat stress conditions. Furthermore, heritability alterations for health and production traits in different cow lactation periods (Van der Spek, van Arendonk, and Bovenhuis 2015; Poppe et al. 2021) might be due to physiological stressors and energy deficiency in early lactation.

From an intergenerational perspective, also non‐genetic inherited factors (e.g., epigenetic effects) may explain differences in direct heritabilities for calf diseases between F0 dam groups (i.e., healthy versus diseased). In HF cows, Kipp et al. (2021b) postulated effects of heat stress on epigenetic modifications, which in causality regulate gene activities and gene expressions, ultimately contributing to genetic parameter alterations. However, a clear discrimination between direct genetic and epigenetic effects implies to model an epigenetic similarity matrix based on whole genome methylation and histone modification data (Thomson et al. 2018). Results from the present study indicate alterations of genetic (co)variance components in dependency of the PMHS, and possible epigenetic effects can be assumed. However, a deeper understanding of intergenerational inheritance and the implementation of respective targeted breeding approaches requires dense epi‐genotyping (Triantaphyllopoulos, Ikonomopoulos, and Bannister 2016; David et al. 2019).

The maternal heritabilities for DIAR and PNEU across PMHS groups in the range from 0.04 to 0.11 were smaller than the respective direct heritabilities. The maternal heritabilities from the present study are in agreement with estimates as outlined in other dairy cattle studies neglecting time‐lagged stressors or specific environmental modelling approaches (Berry 2014; Ring et al. 2019). The moderate direct genetic component plus existing maternal genetic effects, as well as possible epigenetic mechanisms, indicate the complex genetic architecture of calf diseases.

Robertson (1959) suggested a genetic correlation < 0.80 as an indicator for G x E interactions. Accordingly, the direct genetic correlations < 0.80 for the calf diseases DIAR and PNEU between CD_diseased_ and CD_healthy_ dam groups indicate possible G x PMHS interactions. Hence, according to our results, interactions between calf genetic factors and prenatal exposure to a “disease environment” during embryonal or fetal programming might contribute to disease susceptibility in dairy calves after birth. Similarly, studies investigating the effects of in utero heat stress during late gestation reported time‐lagged G x E interactions in offspring (Kipp et al. 2021b). With regard to prompt heat stressor effects on F0 dams directly before birth, Alves et al. (2022) identified G x E interactions for birth weight, average daily gain from birth to weaning and muscling score in F1 beef cattle. The generally stronger effects of prenatal CD than of prenatal MAST grouping on G x PMHS interactions for direct as well as for maternal genetic effects for DIAR and PNEU are unclear. However, in genotype x production system interaction studies, also Shabalina et al. (2021) found smallest genetic correlations between same traits from organic herds with quite large CD prevalence and conventional large‐scale contract herds with a high claw health status.

The strong antagonistic relationships between direct and maternal genetic effects for the calf traits have been confirmed in previous studies. Snowder et al. (2005) reported a pronounced unfavourable direct‐maternal genetic correlation for bovine respiratory disease in beef calves. For calf health traits including omphalitis, diarrhoea and pneumonia, the direct‐maternal genetic correlations were in a range from −0.66 to −0.88 (Yin, Halli, and König 2022). For birth weight, a trait directly expressed after calving and indicating the calf health status, Yin and König (2018b) estimated a negative direct‐maternal genetic correlation of −0.39. Accordingly, in other species, the correlations between direct and maternal genetic effects were of strong antagonistic nature. Aikins‐Wilson, Bohlouli, and König (2021) analysed tail length, tail lesions and growth traits in piglets, and they reported correlations in the range from −0.35 to −0.90. However, biological or physiological reasons explaining the antagonistic associations between direct and maternal effects for health traits or health indicators, are unclear. From a biological perspective, the negative direct‐maternal genetic correlations for DIAR and PNEU indicate that offspring of dams with a maternal genetic ability to produce immunoglobulins are more susceptible to a disease, due to a delayed development of their own innate immune system (Snowder et al. 2005). The complex interplay between active and passive immunity in cattle with effects on calf health has been elaborated by Vlasova and Saif (2021) and should be addressed in ongoing studies in a genetic context. Nevertheless, strong antagonistic relationships between direct and maternal genetic effects for health traits hamper breeding progresses, which might be an explanation for only marginal genetic gain in these traits in the past, as indicated, e.g., for liability in US dairy cattle populations (Guinan et al. 2023). A further explanation for the large correlations between the maternal and direct genetic effects addresses the mathematical perspective in case of small variance components. For example, the direct‐maternal correlation for DIAR was −0.89 (calves from the CD‐diseased dam group). The respective variances were 0.0057 (maternal genetic) and 0.11 (direct genetic). Such parameter constellation implies a negative covariance between direct and maternal genetic effects very close to zero (−0.022). A small change of the covariance towards a positive value close to zero results in a large positive correlation between direct and maternal genetic effects.

### 4.3 Genetic Parameters for Calf DIAR and PNEU on the Continuous Prenatal Herd‐Calving‐Year Prevalence Scale

The direct heritability increase for “DIAR‐CD” at the extreme ends of the herd‐calving‐year scale reflects artefacts from random regression modelling strategies for health traits in previous studies (e.g., Yin et al. 2012). However, we have no convincing argument for the quite strong increase of direct heritabilities for DIAR in dependency of MAST, which differs from the curve pattern along the continuous herd‐calving‐year descriptor for the remaining calf‐dam trait combinations. From our experiences and as observed in some other RRM applications, sporadic genetic parameter curve pattern for specific binary traits might happen. Carlén et al. (2009) applied linear sire RRM to mastitis data which might be “more robust” in case of small disease incidences than animal models, but the artefact of increasing heritabilities and genetic variances at the extreme ends of a continuous time scale was still existent. From a theoretical perspective, Dahlqwist et al. (2019) indicated increased genetic variations with an increasing disease prevalence in human populations. However, also in such theoretical context, it remains unclear why only the prenatal MAST infection status contributed to the quite strong DIAR heritability increases. Twomey et al. (2018) identified only minor heritability alterations for milk production traits in dairy cows with regard to the herd prevalence for parasitic pathogens. Wagner et al. (2021) associated smaller genetic variations for specific mastitis pathogens in “well‐being husbandry systems” with increased genetic variations for health traits under stress conditions. Likewise, Schierenbeck et al. (2011) identified stronger variations of daughter yield deviations for somatic cell count of HF sires in “challenging herd environments.”

With regard to the direct genetic correlations in same traits across different levels for the herd‐calving‐year prevalence, the larger estimates between neighbouring herd‐calving‐year levels compared to levels in greater distance are in agreements with RRM estimates for production traits along continuous time (e.g., Swalve 2000) or climate gradients (Brügemann et al. 2011). However, following the theoretical concept by Robertson (1959), the correlation estimates larger than 0.80 for PNEU disprove any G x herd‐PMHS interactions. In their RRM study, Yin and König (2018a) found stronger genetic correlations declines along herd parameter gradients for low heritability health traits than for moderate heritability production traits. Consequently, and also based on the changes of sire breeding values along the herd scale, they suggested the selection of specific sires for specific herds. In analogy, due to the pronounced genetic correlation decline in the present study for DIAR (minimal estimates of 0.48 (MAST scale) and of 0.78 (CD scale)), herd‐specific sires should be used according to the prenatal herd‐calving year health status. Consideration of the herd‐health status as criterion for herd clustering in across‐country genetic evaluations was suggested by Jaeger et al. (2018) for local dual‐purpose cattle breeds. Pinto et al. (2020) studied cow trait responses for primary and functional traits via RRM along an urbanisation gradient in a tropical country, reflecting the herd hygiene status. Again, breed differences as well as genetic parameter estimates substantially differed for low heritability traits in dependency of urbanisation. Calus and Veerkamp (2003) indicated that the G x E interactions due to alterations in herd averages for BCS and calving characteristics were mostly due to scaling effects. Nevertheless, they observed slight re‐rankings of some sires in the net merit index as a function of the average BCS per herd.

In the RRM analyses, the strongest genetic correlation decline was observed for DIAR along the continuous prevalence scale for MAST. The same calf disease–cow trait combination displayed quite strong heritability alterations. In the bivariate analyses, the smallest direct genetic correlation (0.64) was estimated for PNEU in dependency of the PMHS for CD. In the bivariate model, the sub‐data classification was directly based on the dam PMHS, while the average prevalence within herd‐calving‐years was considered as environmental descriptor in the RRM applications. This might be an explanation for differing estimates from bivariate models and RRM. In agreement with results for the “MAST scale,” the genetic correlation decline was stronger for DIAR than for PNEU on the “CD scale.” Consequently, environmental sensitivity in terms of reactions on the herd health status was more obvious for DIAR than for PNEU. An explanation in this regard might be that diseases which predominantly occur in a narrow interval directly after birth (i.e., DIAR) are strongly influenced by maternal immunity, which is a major component of PMHS. Even stronger indications for time‐lagged G x E interactions on offspring health traits were observed when considering the prenatal stress status in terms of in utero heat stress (Kipp et al. 2021b). In utero heat stress can be interpreted as a component of PMHS, especially in utero heat stress during the last 8 weeks of gestation (Kipp et al. 2021a). Heat stress effects impaired immunocompetence (Bagath et al. 2019). Consequently, Lemal et al. (2023) inferred overlapping genetic mechanisms and physiological pathways for heat tolerance and immune functions in dairy cows, as well as in other species.

## 5 Conclusion

F1 female calves from MAST or CD‐diseased dams had a significantly higher risk for DIAR and PNEU. In the longer time horizon, 305‐d first lactation traits MY, FY and PY of F1 cows were unrelated with the F0 dam health status. Direct and maternal heritabilities for DIAR and PNEU were quite similar with regard to the PMHS of the dam. However, based on genetic correlations smaller than 0.80 for direct genetic effects for calf DIAR and PNEU, slight indications for G x PMHS interactions were observed when creating sub‐datasets according to the CD status of the dam. In RRM applications, quite stable direct heritabilities on the continuous herd‐calving‐year prevalence scale were observed for PNEU. For DIAR, we observed an obvious increase of direct heritabilities and declining correlations for direct genetic effects across the herd‐calving‐year prevalence scale for MAST. The identified G x herd‐PMHS interactions for DIAR suggest consideration of specific sires according to the herd health status for MAST and for CD.

## Conflicts of Interest

The authors declare no conflicts of interest.

## Data Availability

The data that support the findings of this study are available on request from the corresponding author. The data are not publicly available due to privacy or ethical restrictions.
